# Autochthonous Gnathostomiasis in Madagascar

**DOI:** 10.3201/eid2608.200383

**Published:** 2020-08

**Authors:** Annie Raharisoa, Arezki Izri, Romain Lovanirina Andrianjafy, Ranto Andriantsilavina Rajaona, Anthony Marteau, Remy Durand, Mohammad Akhoundi

**Affiliations:** Avicenne Hospital, Bobigny, France (A. Raharisoa, A. Izri, A. Marteau, R. Durand, M. Akhoundi);; Centre Hospitalier Universitaire Joseph Ravoahangy Andrianavalona, Antananarivo, Madagascar (A. Raharisoa, R.L. Andrianjafy, R.A. Rajaona);; IHU Méditerranée Infection, Marseille, France (A. Izri);; Université Paris-Saclay, Châtenay-Malabry, France (R. Durand)

**Keywords:** ocular gnathostomiasis, Gnathostoma spinigerum, zoonoses, parasitic zoonoses, gnathostomiasis, parasites, Madagascar, nematodes

## Abstract

We used molecular tools to identify an autochthonous case of gnathostomiasis in Madagascar. This severe ocular infection, caused by *Gnathostoma spinigerum* nematodes, led to vision loss in the patient’s left eye. Clinicians should be aware of this parasitosis in Madagascar and other countries in Africa.

Human gnathostomiasis is a foodborne parasitic zoonosis caused by spiruroid nematodes of the genus *Gnathostoma* (*1*). There are 13 species of *Gnathostoma*, including 6 from Asia and 7 from the Americas. Of these species, 4 (*G. spinigerum*, *G. hispidum*, *G. doloresi*, and *G. nipponicum*) in Asia and 1 (*G. binucleatum*) in Latin America are pathogenic to humans (*2*). The life cycle of the parasite requires >3 hosts, some of which might be paratenic. Host species vary depending on the *Gnathostoma* species; for most *Gnathostoma* species, humans are accidental dead-end hosts. Ingestion of the third larval stage of *Gnathostoma* spp. in raw or undercooked freshwater fish, eels, frogs, reptiles, or birds results in cutaneous, and sometimes visceral, larva migrans. Other proposed routes of infection include drinking water contaminated with infected *Cyclops* spp. crustaceans and transcutaneous penetration during the preparation of contaminated food (*3*). Clinical signs and symptoms of infection depend on the affected organ(s) (*3*,*4*), which might include the skin; gastrointestinal or genitourinary tracts; lungs; and, more rarely, the central nervous system and the eyes (*5*). Infection results in nonspecific signs and symptoms, such as fever, urticaria, anorexia, nausea, vomiting, and diarrhea, accompanied by larval migration; in the case of central nervous system involvement, infection can be fatal (*3*). Painful or pruritic migratory subcutaneous edema is the most common symptom of *Gnathostoma* infection. Physicians diagnose gnathostomiasis on the basis of eosinophilia, migratory lesions, and the patient’s history of geographic and dietary exposures (*6*).

## The Study

In November 2016, a 24-year-old female farmer noticed an ecchymotic edema in the upper and lower eyelids of her right eye. She lived with her family in a village next to Anjozorobe, a city located 90 km northwest of Antananarivo, Madagascer, and had no history of travel abroad. She had no history of ocular problems or allergic reactions. 

In the following days, the development of conjunctivitis and reduction in visual acuity prompted her to visit the general practitioner. The physician prescribed her a 3-week treatment including several antimicrobial medications. This regimen did not result in clinical improvement. In December 2016, the physician referred her to the Hospital Centre of Joseph Ravoahangy Andrianavalona Ampefiloha (Antananarivo) as a result of her worsening condition.

The interview and clinical examination revealed ptosis, preseptal cellulitis with an abscess, and decreased visual acuity of the right eye. She also had a unilateral headache on the same side (subjective pain rating 4/10). Blood measurements including erythrocyte sedimentation rate, neutrophilic leukocyte count, eosinophil count, and hepatic aminotransferases (aspartate aminotransferase, alanine aminotransferase) were within reference limits. Leukoconcentration and parasitological analysis by microscopy of a thick blood smear did not indicate microfilaria infection. Abdominal ultrasound examination revealed no hepatic or splenic abnormalities. We drained the abscess and prescribed a first-line empirical treatment of gentamicin, metronidazole, and levofloxacin. On the 7th day of hospitalization, the patient reported a sudden pain in the other eye. We conducted a slit-lamp examination, which revealed a worm in the anterior chamber of the left eye. We removed the mobile worm from the left eye through a small sclerocorneal tunnel.

Ocular ultrasound analysis showed a thickened retina. Doppler analysis revealed a hypervascularisation with an inflammatory appearance associated with a retinal detachment or an echodetectable foreign body. Ophthalmologic evaluation of the left eye by funduscopic examination revealed preretinal and vitreous hemorrhages. We prescribed mebendazole (100 mg 2×/d for 21 days) to treat further probable infection by *Gnathostoma* parasites. Unfortunately, the long delay to correct diagnosis and treatment led to blindness in the patient’s left eye.

**Figure 1:**
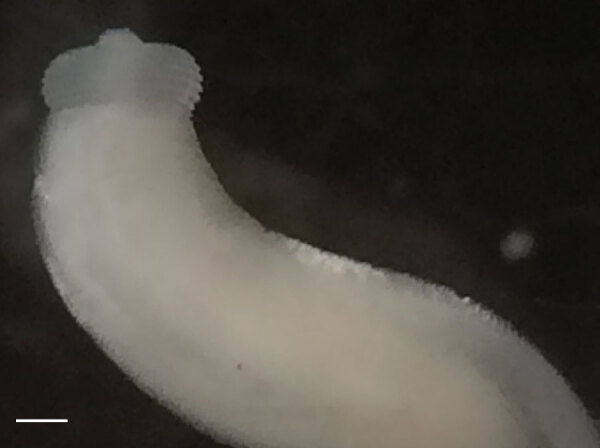
Anterior view of the third-stage larva of *Gnathostoma spinigerum* isolated from left eye of a woman in Madagascar, 2016. Scale bar indicates 1 mm.

Macroscopic examination of the extracted parasite revealed a white cylindrical body 12 mm long and 4 mm wide. Microscopic analysis showed an organism with a bulbous head, a cephalic region covered by transverse rows of cuticular spines, a body curved at the middle, and a shortened anterior end (Figure 1). These morphologic criteria (i.e., body shape, number of rows of hooks at the cephalic end, the spines covering the body) enabled us to identify the worm as a member of a *Gnathostoma* sp. (*3*).

**Figure 2:**
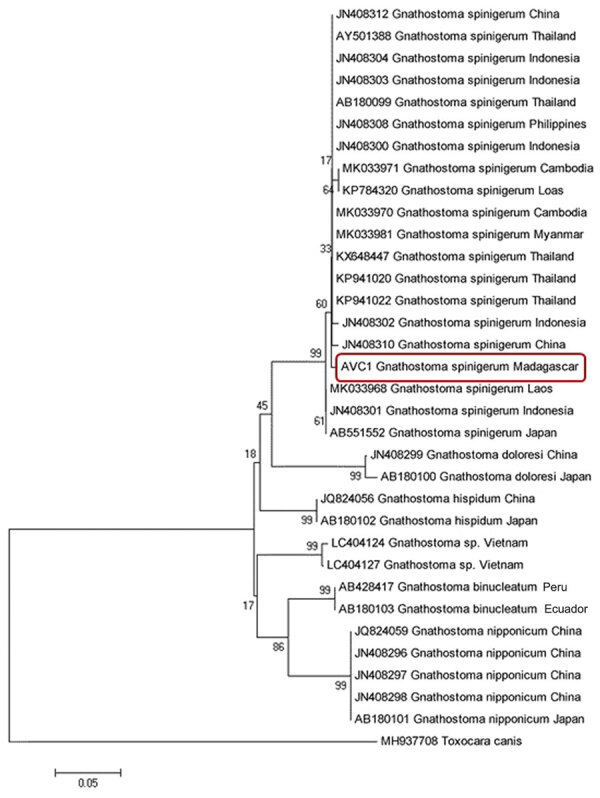
Neighbor-joining phylogenetic tree of *Gnathostoma spinigerum* isolated from a woman in Madagascar, 2016 (red box), and reference sequences from GenBank. The tree was constructed using MEGA (*8*) with bootstrap values determined by 1,000 replicates and compares the cytochrome oxidase I gene sequences.

For further identification, we extracted the DNA of the worm using Chelex 10% (Bio-Rad, https://www.bio-rad.com). We then performed a conventional PCR selective for an 800-bp fragment of the cytochrome oxidase I gene (*7*). BLAST analysis (http://blast.ncbi.nlm.nih.gov/Blast.cgi) identified the specimen as *G. spinigerum* because it shared >99% identity with an isolate from GenBank (accession no. MK033968). We deposited the corresponding sequence in GenBank (accession no. LC505621). We constructed an inferred phylogenetic tree of *G. spinigerum* with GenBank sequences using MEGA version 5.0 software (*8*). Our MEGA analysis used the neighbor-joining method with bootstrap values determined by 1,000 replicates (Figure 2). All sequences, including the one originating from Madagascar, clustered in the same species group. This grouping occurred despite the absence of sequences from other African *Gnathostoma* species.

## Conclusions

Gnathostomiasis is endemic to Southeast Asia, Central America, and South America. A few cases have also been reported from nonendemic regions such as Australia (*9*). Until 2003, this disease was not thought to be endemic in Africa. However, reports of imported cases from Zambia, South Africa, and Botswana ruled out this hypothesis (*5**,**10**–**13*). Furthermore, zoonotic *Gnathostoma* infection has been reported from Zambia (*10*). In 2000, a case of clinical gnathostomiasis was reported in a 43-year-old traveler returning to Italy from Madagascar (*14*).

*G. spinigerum* nematodes, whose primary hosts are cats and dogs, are widely distributed in tropical and subtropical areas, especially in Asia. Consumption of raw meat or freshwater fish in endemic areas is the major risk factor for infection. Consequently, we believe this infection was acquired through eating raw fish or drinking water contaminated by copepods. After being ingested, the larvae probably passed through the gastrointestinal barrier and migrated through the bloodstream to the ocular system. Our patient regularly used the freshwater river near her house for drinking and sanitary purposes. This practice leads us to consider contaminated freshwater, rather than the ingestion of raw fish, as a probable source of her infection. The habit of eating raw meat or fish may explain the higher incidence of *Gnathostoma* infection in some regions of the world, such as Southeast Asia (*7*).

Diagnosing gnathostomiasis can be difficult for physicians who are unfamiliar with this nematode, especially in nonendemic regions. This lack of physician awareness might lead to missed or delayed diagnosis. Physicians should also screen family members of affected patients because many families share eating habits. In this case, we evaluated the patient’s relatives with clinical (checks for painful migratory skin lesions or skin nodules, eye damage) and biological (eosinophil count, erythrocyte sedimentation rate) examinations. We did not observe any signs of infection.

Albendazole and ivermectin are usually considered the best treatments for gnathostomiasis (*1*^2^,*1*^3^). In this case, we prescribed mebendazole according to a local protocol used to cure a wide spectrum of nematode infections.

In conclusion, we used molecular tools to identify a case of *G. spinigerum* in Madagascar. Previous studies in Africa have relied only on morphology for species confirmation. Clinicians should be aware of the existence of gnathostomiasis in Madagascar and other countries in Africa and also of the potentially severe complications associated with ocular gnathostomiasis.
